# Differential range and activity of various forms of the Hedgehog protein

**DOI:** 10.1186/1471-213X-5-21

**Published:** 2005-09-30

**Authors:** Rebecca J Dawber, Stephen Hebbes, Bram Herpers, France Docquier, Marcel van den Heuvel

**Affiliations:** 1 MRC Functional Genetics Unit, Department of Human Anatomy and Genetics, University of Oxford, Oxford, UK; 2 Cell and Molecular Biosciences, University of Newcastle, Newcastle-upon-Tyne, UK; 3 Cell Biology, UMC Utrecht, Utrecht, The Netherlands; 4 Department of Biological Sciences, University of Essex, Colchester, UK

## Abstract

**Background:**

The Hedgehog (Hh) family of secreted proteins act as extracellular messengers to control and coordinate growth and differentiation. The mechanism by which Hh protein travels across a field of cells, and results in a range of specific effects relating to the distance from the source, has been the subject of much debate. It has been suggested that the range and activity of the pathway can be linked to modifications of the Hh protein, specifically the addition of lipid groups at N- and C-terminal sites.

**Results:**

Here we have addressed the potency of different forms of Hh protein by expressing these in *Drosophila*, where we are able to precisely establish pathway activity and range in naïve but responsive tissues. As expected, a construct that can produce all forms of Hh recapitulates endogenous signaling potencies. In comparison, expression of a form that lacks the cholesterol moiety (HhN) leads to an extended range, but the product is less effective at inducing maximal Hh responses. Expression of a point mutant that lacks the N-terminal palmitate binding site shows that the palmitoylation of Hh is absolutely required for activity in this system.

**Conclusion:**

We conclude that the addition of the cholesterol moiety limits the range of the protein and is required for maximal activity, while addition of palmitate is required for all activity. These findings have implications for understanding how Hedgehog proteins move, and thus their potential at influencing distant sites, and concomitantly, how modifications of the signaling protein can affect the efficacy of the response in exposed cells.

## Background

Hedgehog (Hh) proteins are a family of secreted signalling molecules that act as extracellular messengers, signalling between cells to control and coordinate growth and differentiation. Hh proteins govern growth and patterning events in a wide variety of developmental contexts in vertebrates and invertebrates, and mutations in components of the Hedgehog signalling pathway are implicated in many human disorders including cancers [1-4]. In *Drosophila*, the single Hh protein is required at multiple developmental stages and is responsible for patterning embryonic segments [5,6] as well as adult structures such as wings, legs and eyes [7-9]. The response to Hh can vary considerably with the amount of signal received, for example, in the vertebrate ventral neural tube, motor neuron and interneuron generation depends on the graded activity of Sonic hedgehog (Shh) [10]. One key question is how one signal can elicit such a range of responses.

In most cases explored so far in *Drosophila*, Hedgehog transduces its signal through the zinc finger transcription factor Cubitus interuptus (Ci), which can exist in several different forms [11,12]. In the wing imaginal disc, Ci is expressed throughout the anterior compartment [13], in a complementary pattern to *hh *itself. In the absence of Hh signal, through a mechanism which is not fully understood, the Hh receptor Patched (Ptc) prevents Smoothened (Smo) from localising to the plasma membrane [14]. Under these conditions, Ci is present in a complex with a variety of proteins including the kinesin-like protein Costal2, (Cos2) [15,16], the serine-threonine kinase Fused (Fu) [17], and the PEST protein Suppressor of Fused (Su(Fu)) [17]. This complex is associated with microtubules. Through interactions with other proteins including Protein Kinase A (PKA) and the putative ubiquitin kinase Slimb [18,19], Ci is cleaved to generate a 75 kDa transcriptional repressor, which represses transcription of target genes including *hh *and *decapentaplegic *(*dpp*) [20]. On binding of Hh to Ptc, Smo relocates to the plasma membrane [14], Fused becomes phosphorylated and the Ci-Cos2 complex is disrupted and dissociates [15]. Full length Ci is stabilized in the cytoplasm and can then be translocated to the nucleus, where it acts as a transcriptional activator of target genes including *dpp *and *ptc*.

The amount of nuclear Ci appears to be tightly regulated through cytoplasmic/nuclear shuttling and degradation. Hh signalling increases the rate of Ci nuclear import [21,22] while rapid nuclear export also plays a major role in controlling nuclear Ci levels [22]. Ci activation, distinct from Ci stabilization and nuclear import, occurs in response to maximal Hh signalling [23-25]. Ci levels are further regulated by the action of Debra, which mediates the polyubiquitination of full-length Ci, leading to its lysosomal degradation [26]. The result of these molecular pathways regulating Ci activity is visualized at the boundary of Hh-expressing and receiving cells. A broad stripe of cells expressing the full length form of Ci is seen close to the anterior-posterior (A-P) boundary; Ci levels are high in the cytoplasm in these cells. However, very close to the Hh expressing cells, where Hh signalling is maximal and hence Ci is maximally activated, shuttling of Ci into the nucleus and its subsequent rapid export and degradation lead to low cytoplasmic levels of Ci.

Hh proteins undergo a variety of post-translational modifications, some of which have been shown to modulate biological activity. The protein is unique in that it can be dually lipid modified: both an ester-linked carboxy-terminal cholesterol moiety [27] and an amide-linked amino-terminal palmitate [28] have been found associated with the Hh signalling protein. Unprocessed Hh protein undergoes auto-proteolytic cleavage under the control of a catalytic domain in the C terminal region, releasing the active amino-terminal domain. Cleavage is accompanied by covalent bonding of cholesterol to the N-terminal moiety [29,30]. This can be followed by addition of palmitate to the amino terminus. This palmitoylation requires the action of the Skinny Hedgehog acyltransferase, also known as Sightless or RASP [31-33]. The efficient addition of palmitate has been suggested to depend on prior addition of cholesterol [34]. Initial experiments using active Shh protein relied on a construct only encoding for the amino-terminal domain in bacteria. This peptide is active in certain assays, but whether it has full range activity is difficult to establish in *in vitro *systems. More recent experiments indicate that this form of Hh, which is not cholesterol modified, has low level Hh activity in vertebrates [35]. It has not been clearly established whether or not this form is palmitoylated.

Despite lipid modification, which one might expect to limit its movement, Hh moves beyond the cells where it is expressed. Hh directly induces expression of Dpp and Ptc in a band of 5–10 cells beyond the boundary of Hh expression [36-38]. There are two to three cells where Ci levels are low due to rapid degradation and an additional five to seven cells where cytoplasmic levels are high. The action of Dispatched (Disp), a protein homologous to Ptc, is required for secretion of Hh from sending cells [39]. Hh signal reception in receiving cells causes up-regulation of Ptc, the receptor protein for Hh, which also sequesters Hh and thus forms a sink for Hh protein. Non-cholesterol modified Hh expressed in *Drosophila *has been reported to have a broader range of Hh action than normal [30]. Contrastingly, in vertebrates, it has been argued that cholesterol modification is required for long range signalling activity [35,40] and Burke et al suggest that normally processed Hh has a greater range of action than unmodified Hh in *Drosophila *[39]. However, their data do not show the effects of cholesterol-modified Hh expression in comparison with unmodified Hh.

In light of this conflicting evidence, we wanted to determine the effects of Hh modifications on Hh movement and efficacy in inducing target gene expression. Further, and related to this, a key question in signal transduction is how a single molecule can elicit a wide range of responses. Does Hh act through a simple gradient-threshold mechanism, or are other functional factors involved? Specifically, in the light of Hh lipid modifications, do different forms of Hh protein play discrete roles in patterning? To address these questions, we generated clones ectopically expressing various differently modified forms of Hh protein and examined their effects on target gene expression and wing disc patterning. We find that un-cholesterol-modified Hh (HhN) has a greater range of action than normally modified Hh. We further show that the palmitoylation step is crucial for Hh activity, and is likely to occur even in the absence of prior cholesterol addition. We also demonstrate that Hh lacking the cholesterol moiety is less effective than normal wild-type Hedgehog at inducing expression of target genes that require the highest levels of Hh. We discuss the implications for Hh signalling and its effects on target tissues.

## Results

We generated clones of cells expressing various different forms of Hedgehog (Hh-FL, HhN, HhCtoS, HhNCtoS) in an otherwise wild-type wing disc, using a Flp-out cassette to drive expression of the constructs [37]. We expect the Hh full-length (Hh-FL) protein to be processed to yield cholesterol- and palmitate-modified N-terminal signalling domain, HhNp (and other forms if these indeed are present in nature), plus the Hh C-terminal moiety. The HhN form generates the N-terminal directly, rather than by cleavage of immature full-length Hh, and will not have cholesterol added, but should still be modified by palmitoylation. In contrast, the HhCtoS form is mutated at the residue where palmitate is added, but should still undergo cleavage and cholesterol addition. Lastly, a HhNCtoS form cannot have either of the lipid modifications.

**Figure 1:**
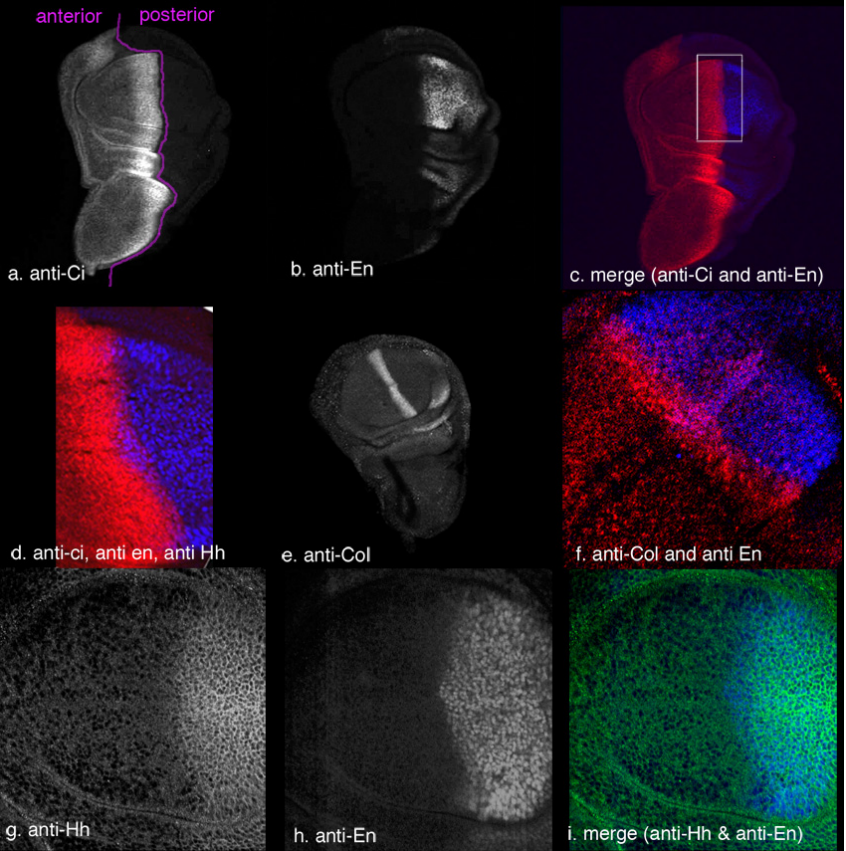
Wild-type expression patterns for Cubitus interruptus, Engrailed, Collier and Hedgehog. Ci in red (c and d), En in blue (c, d, f and i), Col in red (f) Hh in green (i). a. Ci expression; purple line denotes antero-posterior boundary. b. En expression. c. Merge of a and b. d. Enlargement of region equivalent to that boxed in c. Note the overlap between cells expressing En (blue) and Ci (red) at the antero-posterior boundary. e. Collier expression. f. Higher power image showing Col and En. Note the slight overlap at the A/P boundary. g. Hh expression in the wing pouch h. En expression. i. Merge of g and h. It can be seen that there are cells that express En (blue) which do not express Hh (green).

We used various targets of Hh signalling to assess the extent and degree of pathway activation: Engrailed, which requires high pathway activation for maximal induction [9,41], Collier, with a lower threshold for induction [42], and Ci which behaves in different ways depending on the signal intensity, accumulating in response to Hh moderate signal, but showing lowered cytoplasmic levels in response to maximum Hh signal. Figure 1(a–d) shows En and Ci expression; note that at the A-P boundary (purple line), in cells receiving maximal Hh signal, hyperactivated Ci and En are expressed in the same cells (1d). Figure 1(e–f) shows wild-type Collier expression. Notice that Col expression (red) just overlaps En expression (blue) in two cells but extends anteriorly from these in response to lower levels of Hh. Figure 1(g–i) shows Hh and En expression, revealing that an area of En expressing cells exists more anterior to the Hh marked domain: these are the cells producing En in response to high Hh signal, the same set of cells as in overlap of Ci and En in Figure 1d.

We did not use Dpp as a key marker of Hh signal because no antibody is currently available that consistently recognizes the protein. Use of, for instance, *dpp*lacZ inevitably does not reveal as accurate a picture as an antibody, particularly as β-galactosidase shows perdurance in descendants of cells that have previously expressed the construct but have since ceased expression [43].

### Ectopic Expression of full length Hh recapitulates the A-P boundary in the wing disc

**Figure 2:**
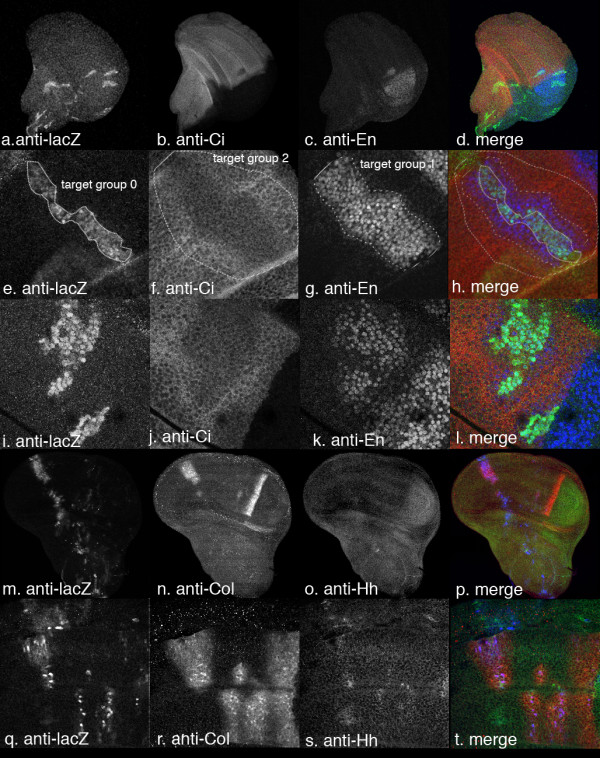
Clones of cells expressing full-length Hh recapitulate the full range of Hh responses. a, e, i, m and q: lacZ, clone marker; b, f, j: Ci; c, g and k: En; n and r: Col; o and s: Hh. D, h, l, p and t: merged images across rows (d, h and l: clone markers in green; En in blue and Ci in red. P and t: clone marker in blue, Col in red and Hh in green). a–d, Ci (red) is down-regulated and En (blue) is up-regulated in and around clones expressing Hh-FL. The anterior pouch of these discs is overgrown (a–d). e–h. Higher magnification of clone similar to those in a–d, showing target cell groups 0 (cells within clone), 1 (2–3 cell diameters immediately outside clone) and 2 (cells further from clone), (see text for details). i–l It can be seen clearly that Ci is down-regulated within and around the clone (in target groups 0 and 1) but is up-regulated further from the clone (target group 2), whereas En is up-regulated in and immediately around the clone (in target groups 0 and 1, but not 2). m–p. Collier is up-regulated in and around clones. It can also be seen that these cells express Hh (green). q–t. Higher magnification images of clones like those shown in m–p. It can be seen that Col is up-regulated in and around the clone (target groups 0, 1 and, at a lower level, 2)

Use of antibodies to detect Hh target proteins reveals that clones of cells expressing Hh-FL, within the anterior presumptive pouch, cause changes in localization and expression of these targets (fig 2). Within the clone, where cells are labelled for LacZ (target group 0, fig 2e), Ci accumulation is reduced (fig 2f,j) and En expression is strongly induced (fig 2g,k). The same decreased Ci and increased En is seen for two to three cell diameters outside the clone (labelled as target group 1 in fig 2g). These cells also activate Collier (Col) expression (fig 2m–t). In cells further from the clone, at 4–8 cell diameters removed (target group 2, fig 2f), Ci accumulation is increased relative to the surrounding cells (fig 2f,j). Col is also expressed in these cells (fig 2r). However, these group 2 cells do not express En (fig 2g,k).

This situation is similar to that seen at the A-P boundary in wild-type wing discs, where cells receiving the highest levels of Hh signal show reduced Ci accumulation (fig 1a–d); this corresponds to the region of hyper-activated Ci. These same cells show En expression activation (see fig 1d). A few cell diameters further from the boundary, there is no En expression, but Hh signal is sufficient here to result in accumulation of full length Ci and to activate *col *expression.

Isolated clones expressing Hh-FL have little molecular effect on the rest of the disc, outside the area as described above. There is a slight increase in Ci levels throughout the anterior of the disc, but the band of elevated Ci near the A-P boundary, a representation of moderate Hh pathway activation, is still clearly visible, indicating the Hh signalling pathway in the remainder of the disc is not (or only very mildly) affected. As expected, clones in the posterior of the wing disc, where Hh is normally expressed, have little or no effect on disc patterning. However, where Hh-FL expressing cells fall within the anterior presumptive pouch and hinge and affect target gene expression as described above, they also cause overgrowth of the disc, with the anterior presumptive wing pouch enlarged compared with a relatively normal sized posterior compartment and presumptive notum.

### Ectopic expression of the Hh amino-terminal fragment induces a distinct set of target genes and at a longer range

**Figure 3:**
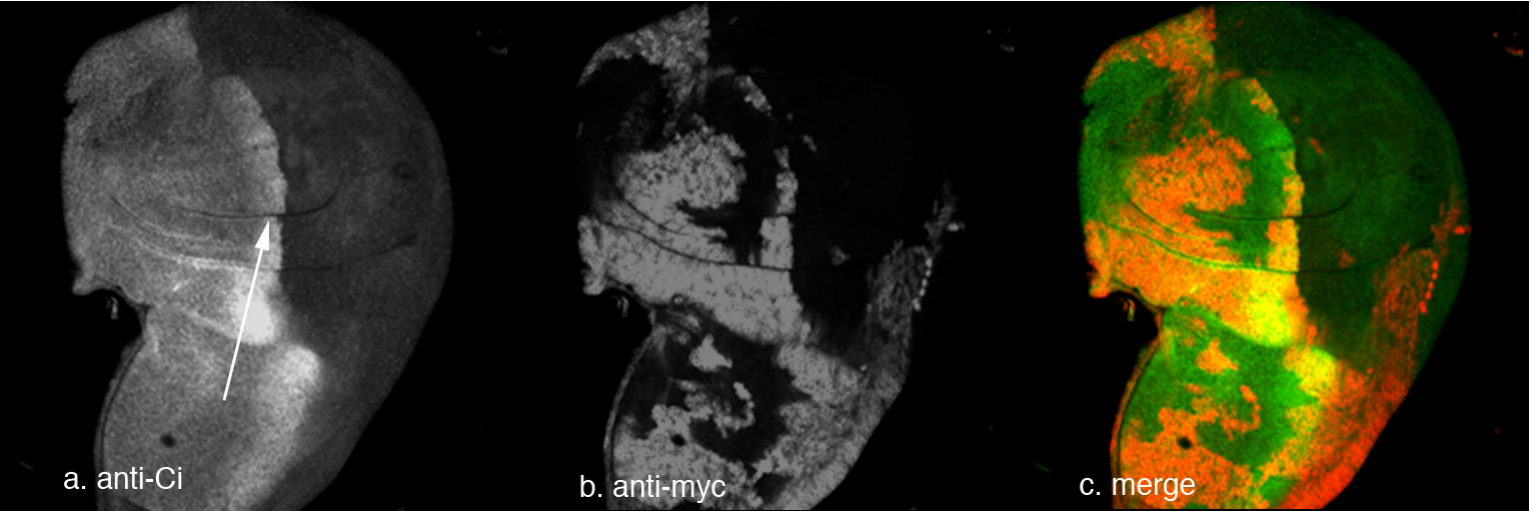
Moderate level Hh signalling despite large *disp *clones. Anti Ci (a) or in green (c), anti-myc (clone marker) (b) or in red (c). Even when large *disp *clones fill almost the entire posterior presumptive pouch (marked by lack of anti-myc staining (b)), some form of Hh is still able to up-regulate Ci accumulation near the A/P boundary (a). Arrow indicates region of Ci accumulation. Note that there is no region of hyper-activated Ci (low level of Ci as shown in Figure 1) in this part of the disc.

The auto-proteolytic cleavage of native Hh protein gives rise to the active N-terminal portion decorated with cholesterol. It is still unclear if a form of Hh exists in nature that has undergone the cleavage reaction but is not decorated with cholesterol. To address this question, we used clonal analysis of the *dispatched *(*disp*) gene. It has been shown that the *disp *gene product is required for the secretion of the cholesterol-modified form of Hh protein [39,44]. By generating large somatic clones mutant for *disp*, we made wing discs where the whole posterior compartment of the wing pouch was *disp *deficient; these cells therefore cannot secrete any cholesterol-modified form of Hh protein. We then analyzed these discs to see if cells, which will not receive cholesterol-modified Hh, can still show any Hh response. In Figure 3, a *disp *clone abutting the A-P boundary contained within the posterior compartment is shown. It is clear that cells in the anterior are able to respond to a Hh signal by generating high cytoplasmic levels of Ci (see arrow, fig 3a). However it is also clear that no maximal response of Hh signalling is generated, as the hyperactivated Ci band directly anterior to the A-P boundary is not seen. Therefore, it appears that although these cells are not receiving any cholesterol-decorated Hh, they are still able to respond to a form of Hh which can be secreted by *disp *mutant cells. This is likely to be a form of Hh that lacks the cholesterol moiety.

**Figure 4:**
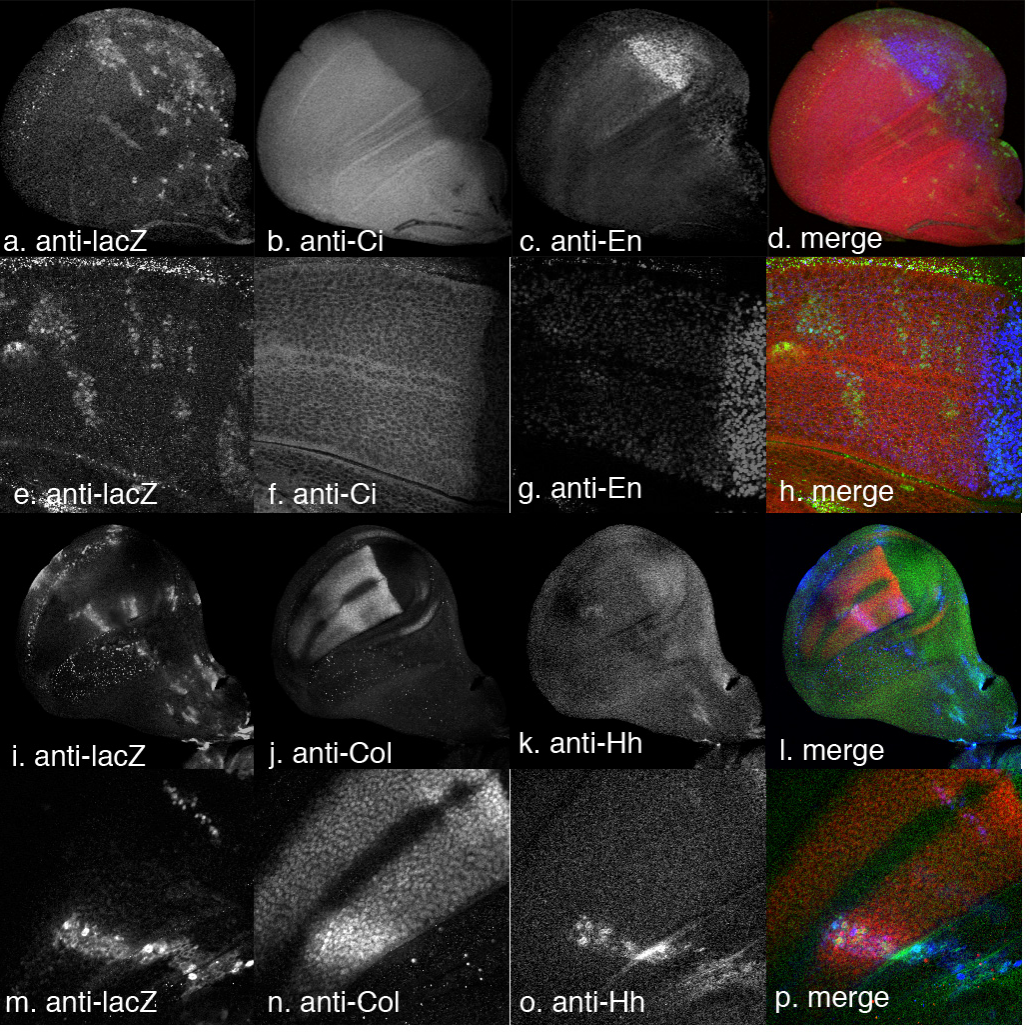
Clones of cells expressing HhN, which lacks the cholesterol moiety, generate moderate Hh responses, over an increased range. A, e, i and m: clone marker (LacZ); b and f: Ci; c and g: En, j and n: Col; k and o: Hh. D, h, l and p: merged images across rows (d and h: clone marker in green, Ci in red, En in blue; l and p: clone marker in blue, Col in red, Hh in green). a–d Ci is up-regulated ubiquitously in disc containing HhN expressing clones, so that the normal strip of elevated expression near the A/P boundary is lost. The entire anterior part of the disc is overgrown (compare with fig 1a–d). e–h. Higher magnification of clones. There is no local effect on Ci (no change within target groups 0–2) although Ci expression is high generally. En is weakly up-regulated in most of the wing pouch. There is very little up-regulation confined to the target group cells. i–l Col is up-regulated in and around clones but expression also extends in two broad stripes through much of the pouch. It can also be seen that the clone cells do express Hh. m–p. Higher magnification images of clones like those shown in i–l. It can be seen that Col is up-regulated in and around the clone (target groups 0, 1 and 2) but is also up-regulated in broad domains extending away from clones. These domains encompass much of the anterior wing pouch but exclude the wing margin.

In order to analyse the downstream effects of a form of Hh that lacks cholesterol, we generated clones of cells expressing a construct that can only produce the cleaved amino-terminal domain of Hh protein (HhN). Ectopic HhN expression within the anterior compartment of the wing disc does not lead to lower levels of Ci in or just outside the clone (fig 4b,f). HhN-expressing clones cause some activation of En within and around the clone (fig 4g), but at a much lower level than that seen with Hh-FL-expressing cells. Importantly, while En is only very mildly up-regulated within clones of cells expressing HhN, the effect extends much further away from the clone than with HhFL, causing two broad but weak stripes of En to extend throughout the anterior pouch in many discs (fig 4c,g). In addition, across the whole disc, Ci levels are dramatically increased (fig 4b,f), so that the band of increased Ci expression normally present at the A-P boundary can no longer be seen (compare Figure 4 with Figure 1). To analyse these apparent long-range effects further, we studied expression of the target of Hh signalling which is normally activated in the area of moderately high Hh signal and high Ci accumulation – Collier – in the anterior pouch area. In this case, the difference between Hh-N and Hh-FL expressing clones is even more obvious. The presence of even quite small groups of HhN-expressing cells in the anterior causes Col expression to extend in two strong stripes through most of the anterior pouch (fig 4j,n).

As with full length Hh, clones of cells expressing HhN in the posterior compartment of the wing disc have no obvious phenotypic effect. Clones expressing HhN in the anterior, like those expressing Hh-FL, cause overgrowth. However, the overgrowth, whilst still confined to the anterior, is often more extensive, and includes the presumptive hinge and notum, and therefore covers the full anterior disc.

Thus it appears that Hh-FL is able to produce the most extreme transformation of cell identity, from anterior to posterior, and recreate maximal Hh signalling, however it can do so only at limited distance. In contrast, HhN is only able to induce lower-threshold responses, but is able to do so at a greater distance from the source.

### Palmitate modification of Hh is required for function

The mature form of Hh is unique in being dually lipid modified; in addition to the ester-linked carboxy terminal cholesterol moiety [30], there is also an amide-linked amino-terminal palmitate [28]. It has been shown that palmitate addition is required for maximal Hh activity [45,46]. To investigate the requirement for palmitoylation, we created a transgene that drives expression of a modified Hh protein in which the critical Cysteine residue, the attachment site for the palmitate, has been altered into a Serine residue. The resulting Hh-CtoS protein cannot be palmitoylated, but theoretically should still be cleaved and modified by cholesterol addition.

**Figure 5:**
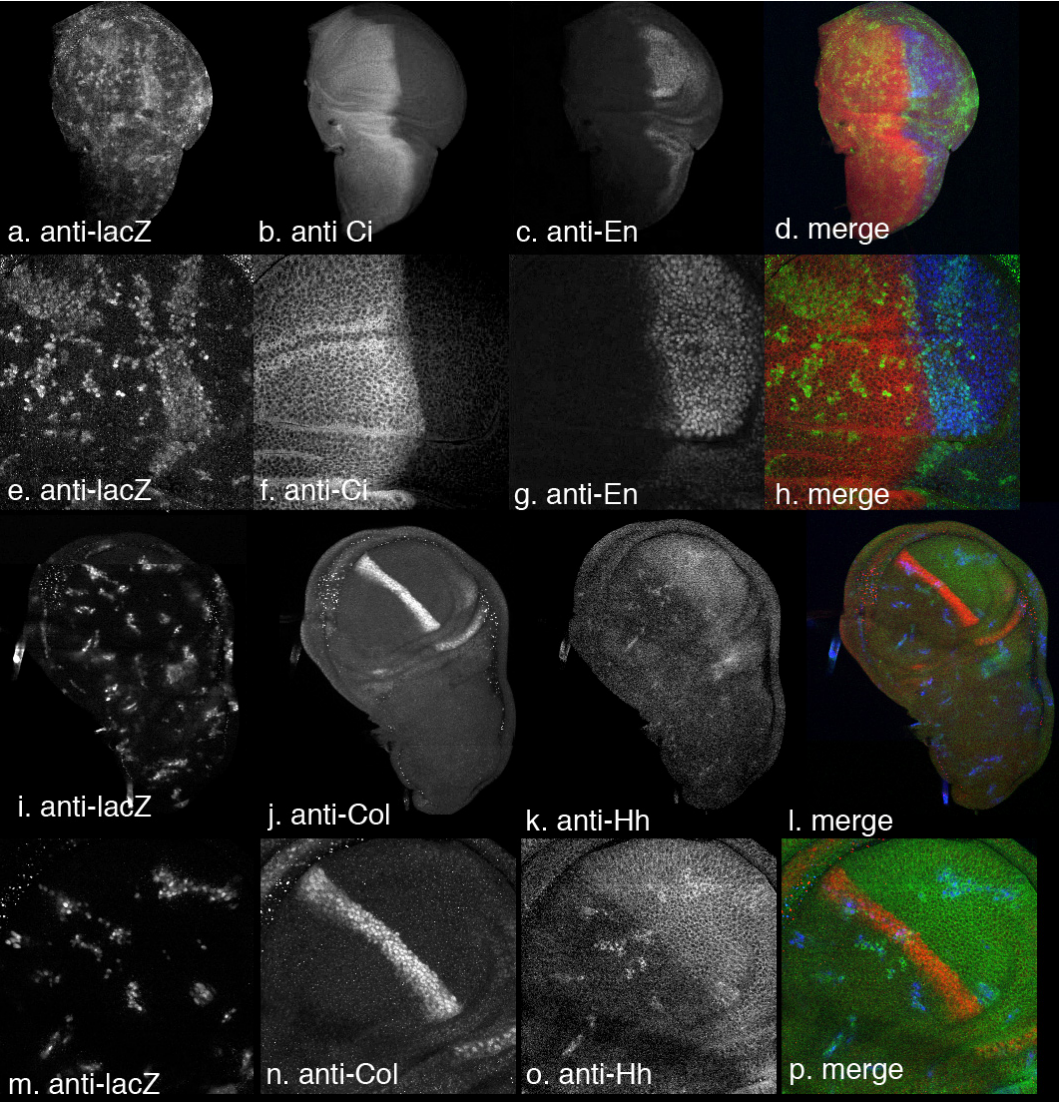
Ectopic expression of HhCtoS has no effect on growth or on any of the markers tested. A, e, i and m: clone marker (LacZ), b and f: Ci, c and g: En, j and n: Col; k and o: Hh. D, h, l and p, merged images across rows (d and h: clone marker in green, En in blue, Ci in red; l and p: clone marker in blue, Hh in green and Col in red). a–d, Clones have no effect on growth, on Ci or on En levels. e–h. Higher magnification of clones. Ci and En expression across disc is normal. i–l Col is unaffected by clones, although they do express Hh (green). m–p. Higher magnification images of clones like those shown in i–l.

Expression of Hh C-S has no discernable effect on Ci accumulation or on expression of the Hh target genes *en *and *col *(Fig. 5). There is no effect on growth or disc morphology. We have shown, using anti-Hh antibody, that the clonal cells do indeed express Hh (Fig. 5k,o). Thus palmitate addition is clearly required for signalling by Hh in this assay.

To determine whether this requirement was linked to cleavage of Hh, we also expressed a form of HhN terminal portion that could not be palmitoylated (HhNCtoS). Ectopic expression of the double mutant HhNCtoS has no effect on disc growth, patterning or expression of target genes, confirming that the addition of palmitate is crucial for function (data not shown). Moreover, the fact that this form has no activity, whilst the HhN mutant is active, suggests that at least some of the HhN as expressed in this study must be modified by palmitoylation. If it were not, HhN would be predicted to behave similarly to HhNCtoS.

## Discussion

There has been a debate in the literature as to whether addition of cholesterol increases or decreases the efficacy of the Hh signal. Much of the conflicting data has been obtained in vertebrate systems, where there are complications depending on whether the tissue tested has already been exposed to native Hh proteins, and whether it is competent to show the full range of responses. The advantage of using *Drosophila *imaginal tissues for this analysis is that it is possible to express Hh forms in tissue that is naïve, i.e. is not itself generating or receiving Hh, but which is fully responsive. Our results show that ectopic expression of a construct able to encode full-length wild-type Hh protein results in the full range of Hh responses. In contrast, expression of a construct encoding Hh lacking the cholesterol moiety (HhN) mimics moderate Hh signal, and is unable to reproduce maximal responses. Further, and importantly, HhN has a much longer range than wild-type Hh when expressed under the same conditions. We also show that palmitoylation is required for activity, both for the cholesterol-modified form of Hh and for HhN. The implications of these findings are considered below.

### Palmitate is required for activity

Our results show that palmitoylation of Hh is required for activity. The CtoS mutation replaces the N-terminal cysteine residue, where the palmitate residue is attached, with a serine residue, disabling the modification. Expression of HhCtoS in wing imaginal disc tissue has no effect on disc growth, shape, or on expression of any of the markers for Hh signalling tested. We have shown that the construct does indeed express Hh, as indicated with anti-Hh antibody, but it has no activity. Thus palmitate is absolutely required for efficacy of Hh signalling in imaginal tissues. This is supported by the finding that the product of the *skinny hedgehog *gene, *ski *(also known by other names), responsible for palmitoylation of Hh, is required in Hh-expressing cells for production of Hh signalling activity and patterning in both embryos and in imaginal discs [31-33]. Similar results to those shown here have been obtained previously [47].

Further, we find that the double mutant HhNCtoS has no activity, whilst HhN displays a subset of Hh functions, suggesting that HhN must possess the palmitate (or similar) moiety, otherwise it would be inactive as is the double mutant. This somewhat contradicts previous work which suggested that cholesterol is required for subsequent efficient addition of palmitate [34]: our results indicate that activity is dependent on the residue where palmitate is added, regardless of cholesterol modification. The exact role of palmitate is unclear. It may be required for efficient binding of Hh to its receptor Ptc, alternatively, it could be required earlier for secretion of Hh from the producing cells. Our results are consistent with recent findings on the vertebrate Hh proteins [46].

### Cholesterol and activity

Our results demonstrate that cholesterol modification of Hh is required for maximal signal activity. We believe that we use the best possible criteria for defining maximal versus moderate activity of Hh signal, namely those found naturally in the wing disc. We define maximal activity in terms of two main criteria. One is strong activation of En, which is well documented as a response to highest Hh signal and which occurs normally in the cells at the A/P boundary [9,41], which do not themselves express Hh but will receive the highest possible Hh signal; these cells express Ci (see fig 1). The other criterion is lowered levels of cytoplasmic Ci in regions where Ci normally accumulates, which has also been shown previously to occur in cells receiving maximal Hh input [21,22,48] and, again, which occurs naturally in cells at the A/P boundary (fig 1). This effect is believed to be a result of Ci hyper-activation, resulting in rapid nuclear import and subsequent degradation. Ectopic expression of wild-type Hh, which will result in production of cholesterol- and palmitate-modified HhNp (plus other naturally occurring forms), results in strong induction of En and lowered cytoplasmic Ci levels within and around the clone, whereas ectopic expression of HhN, which lacks cholesterol, only causes very weak induction of En and high accumulation of Ci in the cytoplasm.

This requirement for cholesterol for maximum efficacy of Hh signalling could be due to a number of factors, the most obvious being more efficient binding and/or inactivation of the Hh receptor Ptc or higher concentration of Hh near the producing cells. The first is an attractive hypothesis, as Ptc contains a sterol-sensing domain [49], involved in binding cholesterol and shown to be important in vesicle trafficking and protein localisation (reviewed [50]). Thus cholesterol-modified Hh may bind to Ptc with higher affinity than HhN, and indeed this has been suggested for Shh *in vivo *[51]. Alternatively, cholesterol-modified Hh might bind Ptc with similar efficiency to HhN but be more efficient at 'inactivating' Ptc, allowing increased activation of Smo and hence of the signalling pathway. The other attractive hypothesis is that cholesterol-modified Hh may be more concentrated near the producing cells than is its cholesterol-lacking relative, either because it is more 'sticky', tending to remain bound to the membrane of producing cells; this could be due to Hh's binding to proteoglycans and Shifted (see [52,53] and [54]). Alternatively, this could be due to cholesterol-decorated Hh associating more efficiently into lipid-rich multi-molecular particles. The presence of such particles containing Hh, and also containing lipophorins, the fly homologue of lipoproteins, has recently been demonstrated [55]. Given the lipophilic nature of these complexes and their similarity to lipoproteins, it is likely that cholesterol would promote association of Hh with these particles, increasing its concentration locally and perhaps also its signalling efficacy. Thus cholesterol addition may increase the potency of Hh signal by concentrating Hh near the producing cells or via increased interaction with Ptc through its sterol-sensing domain or through a combination of the two.

### Cholesterol and range

HhN that lacks the cholesterol moiety travels further from the producing cells than normally processed Hh. This is most clearly seen by the broad stripes of Col activation induced through much of the anterior pouch in response to even small clones of HhN producing cells. This is in marked contrast to the effects of Hh-FL expressing clones, which only induce Col in and around the clone. It is further demonstrated by the high ubiquitous levels of Ci seen throughout the anterior pouch in discs expressing HhN; the native stripe of elevated Ci is impossible to distinguish in these discs. En is only very weakly activated in response to ectopic HhN expression, yet En too appears in two broad stripes occupying much of the presumptive anterior wing pouch, albeit at very low levels. We also observe similar weak but ubiquitous expression of Dpp (data not shown), with HhN expressing clones; while HhFL expressing clones activate Dpp in, and immediately around, the clone (data not shown; all assayed using dppLacZ). This longer range of travel of Hh in the absence of cholesterol may be due to the same reasons that cholesterol-decorated Hh is more efficient at inducing maximal signalling responses; namely, either that cholesterol increases binding affinity to Ptc or that it serves to concentrate Hh near the producing cells. If cholesterol facilitates binding to Ptc, then in its absence Hh would be expected to travel further before eventually binding Ptc and being internalised; if cholesterol serves to concentrate Hh near the producing cells then lack of cholesterol will mean that it can diffuse or travel further from its source.

### Cell proliferation

Hh proteins have been shown to influence growth directly through control of cyclins [56,57]; however indirect control via target genes, which in their turn influence proliferation, is also required for proper tissue growth and patterning [58,59]. We observe a difference in cell proliferation in the wing induced by HhN as compared to HhFL, and we propose that this difference may be due to a difference in the ability to induce downstream targets. Wing discs containing clones expressing HhFL show strong overgrowth in the anterior pouch, but this overgrowth is largely confined to the pouch, having little effect on presumptive hinge and notum regions. In contrast, disc with HhN expressing clones show overgrowth throughout the anterior, including the notum, and generally show a greater degree of overgrowth. It has been shown that *dpp*, a direct transcriptional target of Hh, induces proliferation [59]. By examining the effects of Hh expression on *dpp*-lacZ (data not shown) we observe that only HhFL can induce strong *dpp *expression in and around the clone; HhN induces *dpp*-lacZ more weakly, but does so throughout the whole anterior compartment (pouch and notum). Therefore we propose that the difference in overgrowth is due to far-ranging Hh possibly directly inducing growth in HhN expressing discs, whereas localised activation of Dpp may contribute to the more limited overgrowth in the case of HhFL. These observations have some implications on tissue overgrowth induced by Hh proteins. The question of whether tumour growth is a direct consequence of Hh itself or an indirect effect via other target genes might be relevant for treatment objectives.

### Hh signalling: gradient or different Hh forms?

The work presented here raises some issues as to how Hh signalling exerts its effects across a field of cells. Does Hh signalling function within the definition of a simple concentration gradient or elicit a range of responses via a combination of different Hh forms contributing to different aspects of patterning? For this mechanism to work, it would imply that HhN exists *in vivo*. One piece of evidence suggests that it does. *Disp *has been shown to be required for secretion of cholesterol modified Hh [39], whereas HhN can be secreted even in the absence of *disp *[44]. We show here that large *disp *mutant clones, which would therefore be expected to prevent any secretion of cholesterol-decorated Hh, abolish all maximal Hh response at the A/P boundary (there is no longer a stripe of lowered Ci at the A/P boundary), however, discs containing such clones still show moderate Hh responses, i.e. elevated Ci close to the A-P boundary (see fig 3), very similar to the level of response seen when HhN clones are induced. As no cholesterol-modified Hh will be secreted from the *disp *clone, this moderate Hh response is presumably due to secretion of HhN although this is indirect evidence based on the response only.

Therefore we would like to propose that in nature a non-cholesterol-modified form of Hh is made and this participates in complexes with the cholesterol-modified form in creating a gradient of Hh activity. We postulate that there may be complexes with different ratios of the two forms. The cholesterol form reduces the range of these complexes but induces highest activity. Complexes with more cholesterol-modified Hh will have short range but induce maximal responses at the A-P boundary, whereas those with increased amounts of HhN will be responsible for the more moderate response further from the source of Hh. It is interesting to note here that clusters of Hh protein have been shown to exist in nature [46]. The different modifications of Hedgehog protein might affect its presence in particular clusters.

## Conclusion

There has been some debate in the literature as to the role of cholesterol in Hh signalling: by expressing differing forms of Hh in tissue which is both naïve in terms of previous Hh signal and responsive to that signal, and by careful choice of markers to examine signalling levels, we believe that we have the best possible system to address this dilemma, and we find that cholesterol potentiates the ability of Hh to induce maximal response, while limiting its range. Our results indicate that the morphogen-like behaviour of Hh protein is not solely dependent on a concentration-led induction of activity, but rather might be the result of different forms of Hh protein with differing activities and diffusion rates signalling across a field of cells.

## Methods

### Fly strains

Flp-out constructs are as described by Basler and Struhl (1994). Crosses were of the general form HsFlp/HsFlp; UAS-*hh *× actin>*y*^+^>Gal4 UAS lacZ, or HsFlp/HsFlp; UAS-*hh *× actin>*y*+>Gal4 UAS ubiGFP (> depicts FRT sites).

X-chromosomal HsFlp, actin>*y*^+^>Gal4 UAS-lacZ, and actin>*y*+>Gal4 UAS-GFP containing stocks were obtained from Bloomington. A single heat-shock was delivered to larvae in plastic vials submerged in a 37°C waterbath for one hour in second larval instar (approximately 3 days after egg laying). UAS-*hh*FL, UAS-*hh*N and UAS-*hh*CtoS constructs were generated by BH using standard PCR and specific primers to generate the mutant constructs. Transgenic lines were obtained in this laboratory. The FRT82B *dispatched*^377 ^chromosome stocks were obtained from K Basler.

*Dpp*-lacZ is an insertion of the Exelexis P[*dpp*-lacZ.Exel1.2] stocks, in which the *dpp *disk enhancer described in St. Johnston et al., 1990 [60] drives lacZ expression. This is a homozygous viable and fertile insertion on the third chromosome, obtained from Bloomington.

All flies were reared at 25°C and fed on standard media.

### Generation of Hedgehog constructs

Products of PCR amplified *hh *fragments were cloned into pGEM-T (Promega) and sequenced to verify the constructs. The sequences were then cloned into the pUAS vector and transgenic lines generated.

Full length *hh *was obtained as clone and used as template for the following PCR reactions. The primers we used for PCR were (all shown 5' to 3'):

5'hhN: ATGGATAACCACAGCTCA (used to generate all *hh *constructs)

3'hhC: TCAATCGTGGCGCCAGCT (used to generate full length coding sequence)

3'hhN CtoS: GCATGCCAGTCCGGACAC (reverse primer to generate 5' end of *hh *for CtoS mutation)

5'hhN CtoS: CACAGCTCTGGTCCCGGGCGA (forward primer to generate 3' end of *hh *for CtoS mutation)

3'hhN:CTCGAGTTAGCCGTGCACGTGG (used to generate hhN constructs at 3' end).

### Antibody staining of imaginal discs

Dissection, fixation and antibody stainings were performed as follows. Larval heads were cut off in 1 × PBS on ice. They were inverted and fixed twice in 1 × PBS, 4% paraformaldehyde, firstly for 20 minutes on ice, then following 2 washes in 1 × PBS, fixed again for ten minutes at room temperature. The inverted heads were then washed five times with 1 × PBS and washed a further five times for 5 min each with 1 × PBS, 3% normal serum, matched to the secondary antibodies, and 0.5% saponin (PBT). They were incubated overnight at 4°C with the primary antibodies in PBT and washed again five times with PBT, the next day. Secondary antibodies were used at the manufacturer's recommended concentrations for 2 hr in PBT at room temperature, after which the heads were washed again five times with PBT. Discs were dissected from the heads in PBT and mounted in VectaShield (Vector Laboratories) on glass slides.

Primary antibodies were obtained as follows: Anti-Collier antibody was obtained from M. Crozatier, anti-Hh antibody from I. Guerrerro, anti-Engrailed, 4F11, from N. Patel and anti-Cubitus interuptus, 2A1, which recognises full length Ci, from R. Holmgren. Mouse anti-B galactosidase antibody was from Promega, rabbit anti-B Galactosidase, Rabbit and Goat anti-GFPs were from Abcam. Secondary antibodies were either FITC, TRITC and Cy5 labelled, from Jackson ImmunoResearch, raised in donkey, or Alexa-labelled from Molecular Probes, raised in goat.

Confocal microscopy was performed on a Zeiss LSM Meta-510 and images were processed with the accompanying Zeiss software and in Adobe Photoshop.

## Abbreviations

A-P – antero-posterior

Ci – Cubitus interruptus

Col – Collier

*disp *– *dispatched*

*dpp *– *decapentaplegic*

En – Engrailed

Fu – Fused

Hh – Hedgehog

HhFL – full-length Hh

HhN – N-terminal Hh, lacking the cholesterol moiety

HhCtoS – Hh with the cysteine residue, required for palmitoylation, mutated to a serine

HhNp – normally processed Hh N-terminal region

PKA – Protein Kinase A

Ptc – Patched

*ski *– *skinny hedgehog *(also known as *sightless *or RASP)

Smo – Smoothened

Su(fu) – Suppressor of fused

Wg – Wingless

## Authors' contributions

RJD carried out the genetics, immunohistochemical studies and drafted the manuscript. SH and BH generated and injected the constructs. FD performed the Dispatched analysis and MvdH conceived the study, and participated in its design and coordination.
